# CeO_2_ Nanoparticles Reduce Oxidative Stress and Delay the Degeneration of Intervertebral Disc

**DOI:** 10.1155/bca/3399767

**Published:** 2025-07-13

**Authors:** Sheng-Jie Chang, Xin-Wei Zhang, Hao-Wei Xu, Shu-Bao Zhang, Xiao-Wei Liu, Yu-Yang Yi, Wei Pan, Kai Li, Shan-Jin Wang

**Affiliations:** ^1^Department of Spinal Surgery, Shanghai East Hospital, School of Medicine, Tongji University, Shanghai 200092, China; ^2^Key Laboratory of Inorganic Coating Materials CAS, Shanghai Institute of Ceramics, Chinese Academy of Sciences, Shanghai, China; ^3^Center of Materials Science and Optoelectronics Engineering, University of Chinese Academy of Sciences, 19 Yuquan Road, Beijing, China

**Keywords:** cell senescence, intervertebral disc degeneration, nano-enzyme, PEG-CeO_2_NPs, ROS

## Abstract

The oxidative stress of the body can destroy the homeostasis and lead to a series of adverse outcomes. In recent years, nano-enzyme materials, as a new hotspot in materials science, have been gradually applied in various fields because of their enzyme-like activities at the nanoscale and their ability to regulate various physiological processes in organisms. In this study, we developed a novel cerium oxide (CeO_2_) nano-enzyme drug and demonstrated that the nano-enzyme can effectively improve oxidative stress levels and delay disc degeneration in rats. The experimental results confirmed that in in vitro experiments, the novel cerium oxide nano-enzyme could significantly reduce the ROS level in cells, delay cell senescence, reduce the level of apoptosis, and improve the metabolic state of nucleus pulposus cells. At the same time, it maintains low toxicity to cells. At the animal level, imaging and histomorphological evaluation showed that the novel cerium oxide nano-enzyme could significantly improve the disc height index, MRI Pfirrmann grade, and histological grade scores in rats. In summary, we have developed a successful cerium oxide nano-enzyme, which can be used to reduce the degeneration level of intervertebral disc and provide a new potential idea for clinical treatment of patients with lumbar disc herniation.

## 1. Background

Low back pain (LBP) has brought great troubles to human health around the world, among which the intervertebral disc degeneration (IVDD) is considered to be one of the main causes of LBP [1]. When IVDD occurs, it undergoes a series of biochemical, cellular, and anatomical changes [2], which eventually lead to the rupture of the annulus fibrosus (AF) and the prolapse of the nucleus pulposus (NP) and cause LBP. At the cellular level, IVDD can be manifested as the increase of cellular oxidative stress and apoptosis. At the extracellular matrix level, when IVDD occurs, nucleus pulposus cells (NPCs) secrete less type II collagen and instead secrete more matrix metalloenzymes (MMPs) that break down extracellular matrix [3]. At present, there is still no effective treatment for disc degeneration, so it is urgent to explore new means and new methods to delay disc degeneration.

Reactive oxygen species (ROS) is a class of highly active molecules containing oxygen, including singlet oxygen (^1^O2), hydrogen peroxide (H_2_O_2_), superoxide (O2^•—^), and hydroxyl radical (•OH) [4]. Many inflammatory reactions and degenerative diseases in human body are related to the excessive production of ROS in the state of oxidative stress [5]. Many studies have proved that IVDD is highly correlated with the disorder of ROS dynamic balance [2, 6, 7]. The removal of ROS in cells mainly depends on the rapid reaction of antioxidants and antioxidant enzymes, such as superoxide dismutase (SOD), glutathione peroxidase (GPx), catalase (CAT), and glutathione reductase (GR) in physiological conditions [8], but these enzymes have short half-lives and cannot continuously and effectively remove ROS. Therefore, it may be a good idea to synthesize stable and efficient nano-enzyme drugs with long half-life to remove ROS.

Cerium (Ce) is an active rare earth element, which exists in nature mostly in the form of oxides. The oxide of cerium has two valence states, +3 and +4, and the conversion between the two valence states provides its REDOX ability. At the same time, its significant oxygen vacancy defect promotes the REDOX process and catalytic activity [9]. CeO_2_ has strong antioxidant capacity and free radical scavenging ability [10], which can correct the abnormal REDOX state in cells, reduce the level of oxidative stress in the body, and thus reduce inflammation. Cerium oxide can be administered in various ways, including oral, intravenous, direct intervertebral disc injection, and combined with other nanocarriers [11, 12].

In this study, we synthesized low-molecular-weight PEG (PEG-600)–coated CeO_2_ nanoparticles (NPs). In the description that follows, we will refer to these particular NPs as PEG-CeO_2_NPs. PEG-CeO_2_NPs were evaluated in vitro for their ability to clear ROS, reduce cell apoptosis, correct mitochondrial potential, resist cell aging and proliferation damage, and improve the synthesis and metabolism of NPCs. The therapeutic effect of PEG-CeO_2_NPs in a rat model of intervertebral disc acupuncture was also evaluated. By synthesizing a safe and effective nano-enzyme, this study provides a new drug treatment idea for the treatment of IVDD.

## 2. Materials and Methods

### 2.1. Preparation of PEG-CeO_2_NPs

PEG-CeO_2_NPs were prepared by alkaline treatment. Specifically, cerium (III) nitrate hexahydrate (Ce(NO_3_)_2_·6H_2_O) was added into PEG solution (1 wt%/vol) to reach a concentration of 25 mM. To start the precipitation reaction, ammonium hydroxide (2 M) was added dropwise at a rate of 0.1 mL/min under continuous stirring to adjust the pH of solution to 10. After 4 h of reaction at room temperature, the precipitate was completely formed. The obtained NPs were centrifuged at 8000 rpm for 15 min and washed three times with deionized water. The resulting NPs were denoted as PEG-CeO_2_NPs, dispersed, and stored in deionized water. NPs prepared with Ce(NO_3_)_3_ solution without PEG served as the control group and were labeled as CeO_2_NPs.

### 2.2. Characterization

The microstructure of the NPs was observed using transmission electron microscope (TEM, JEM-2100F, JEOL, Japan). X-ray diffractometer (XRD, D/max-2550, Rigaku, Japan) was employed to analyze the phase composition of PEG-CeO_2_NPs. Fourier transform infrared spectrometer (FTIR, Thermo Scientific, USA) was applied to analyze the presence of PEG. The surface electronegativity of PEG-CeO_2_NPs was measured by Zetasizer Nano ZS NP analyzer (Malvern, UK). Thermogravimetric analysis (TGA) was conducted by the thermal analyzer (NETZSCH STA449C, Germany) to determine the content of PEG. For the characterization of the elemental composition of PEG-CeO_2_NPs and the valence state of Ce, X-ray photoelectron spectroscopy (XPS, ESCALAB-250, Thermo Fisher Scientific, UK) was employed.

### 2.3. Evaluation of ROS Scavenging Activities

The H_2_O_2_ scavenging capacity of PEG-CeO_2_NPs was assessed by measuring the remaining H_2_O_2_ and oxygen generation. PEG-CeO_2_NPs were dispersed in H_2_O_2_ solution (7 mM) at concentrations of 10, 20, 50, and 100 μg/mL. After a 2 h reaction, absorbance of the remaining H_2_O_2_ was measured employing UV-Vis diffuse reflectance spectroscopy (UV-Vis DRS, UV4100, METASH, China) in the range of 200–350 nm. Additionally, PEG-CeO2NPs were added to 10 mL H_2_O_2_ solution (1 mM) at concentrations of 10, 20, and 50 μg/mL. The concentration of dissolved oxygen was monitored in real-time using a dissolved oxygen analyzer (JPB607A, Lei ci, China) under gentle magnetic stirring in a sealed container. The •O_2_^−^ scavenging ability of PEG-CeO_2_NPs was determined by T-SOD assay kit (Nanjing Jiancheng, China). PEG-CeO2NPs at concentrations of 10, 20, 50, and 100 μg/mL were added to the reaction solutions according to the kit instructions. The absorbance at 550 nm was measured by UV-Vis DRS.

### 2.4. Cell Culture

Sprague-Dawley rats (SD rats, either sex) of about 4 weeks of age were injected intraperitoneally with 2% pentobarbital (50 mg/kg), the tail was disinfected, and the lumbar spine was severed. After ethanol soaking, the fibrous ring of lumbar intervertebral disc was cut, the colloidal NP tissue was extracted, 0.2% type II collagenase was added and digested at 37°C for 3 h, and the suspension was filtered with 200-mesh sterile nylon membrane. After centrifugation at 1000 rpm/min for 5 min, discard the supernatant. Complete medium (DMEM/F12 medium containing 10% fetal bovine serum by volume [13]) was added to prepare cell suspension by blowing and mixing and then transferred to 100 mm Petri dish for culture. Cells were cultured in an incubator containing 5% CO2 at 37°C and saturated humidity. After the cells were overgrown, digestion and passage were performed with 0.25% trypsin-EDTA solution. After extracting primary NPCs from SD rats, we increased the concentration of 2% pentobarbital from 50 to 100 mg/kg for intravenous infusion to ensure euthanasia of the rats.

### 2.5. CCK8

CCK-8 kit (C0042, Beyotime, Shanghai, China) was used to detect the effects of H_2_O_2_ or PEG-CeO_2_NPs on NPC activity. NPCs were transferred into 96-well plates with a cell density of about 6000 cells per well. It was co-cultured with different concentrations of H_2_O_2_ (0, 200, 300, and 400 μg/mL) and PEG-CeO_2_NPs (1, 5, 10, 20, and 50 μg/mL) for 24 h (100 μL complete medium). After 10 μL enhanced CCK-8 reagent was added and incubated at 37°C for 30 min, the absorbance was measured at 450 nm wavelength by spectrophotometer. Suitable concentrations of H_2_O_2_ and PEG-CeO_2_NPs were screened for subsequent experiments.

### 2.6. ROS Detection

The ROS concentration was detected using a fluorescent probe of dichlorodihydrofluorescein diacetate (DCFH-DA, S0033S, Beyotime, Shanghai, China). NPC was washed with PBS for two times and cultured in DMEM medium with 10 μM DCFH-DA for 30 min. After washing with PBS three times, capture images under an inverted fluorescence microscope. Average fluorescence intensity was measured using the ImageJ software (Version 1.5.3).

### 2.7. Mitochondrial Membrane Potential

The membrane potential of isolated mitochondria was measured using the JC-1 mitochondrial membrane potential assay kit (C2006, Beyotime, Shanghai, China) according to the manufacturer's instructions. After washing with PBS, NPC and JC-1 dyeing solution were incubated at 37°C for 20 min. Then wash three times with JC-1 staining buffer. Observe the image using an inverted fluorescence microscope and analyze it using ImageJ software (Version 1.5.3).

### 2.8. 5-Ethynyl-2′-Deoxyuridine (EdU) Assay

EdU was measured using the BeyoClick EDU-555 Cell proliferation assay kit (C0075S, Beyotime, Shanghai, China) as per manufacturer's instructions. The cells were treated with H_2_O_2_ or H_2_O_2_+PEG-CeO_2_NPs and incubated with EdU solution diluted with cell culture medium at 1:1000 ratio for 2 h. The cells were fixed with paraformaldehyde. Apollo staining was performed using Apollo staining reaction solution, and DNA staining was performed using Hoechst 33342 reaction solution. After staining the nucleus with Hoechst 33342, images of the specimen were captured using a fluorescence microscope.

### 2.9. Age-Related β-Galactosidase Assay

Senescent cells were stained with senescent β-galactosidase staining kit (C0602, Beyotime, Shanghai, China). Age-related β-galactosidase (senescence-associated beta-galactosidase (SA-β-gal)) activity was measured according to manufacturer's instructions. The NPCs in 6-well plates were washed twice by PBS under different culture conditions. Then, 1 mL SA-β-gal staining fixative was added to each well and fixed at room temperature for 15 min. Discard the liquid and wash 3 times with PBS. Add 1 mL of the prepared dyeing work solution to each hole and leave overnight at 37°C. Images were taken with the microscope (Axio Vert A1, Carl Zeiss, Germany). The quantitative method for fluorescence figures is similar to that for other fluorescence figures, and the quantitative method here is also applicable to other fluorescence figures in this paper. Specifically, the first step is to select the representative staining region, use ImageJ software for cell count, so that the difference in the number of cells in the representative region of each treatment group is not more than 20%, and then count the cells expressing β-galactosidase, that is, the cells containing dark blue substances in the cell body. The proportion of positive cells in the total number of cells in the visual field is the proportion of senescent cells. Each treatment group selected 5 different representative visual fields for statistics, and the experiment is repeated three times.

### 2.10. Immunofluorescence Staining

The P3 generation NPC was transferred to a 24-well plate (3 × 10^4^ cells per well), the supernatant was discarded, and after washing with PBS, the NPC was fixed with 2% paraformaldehyde solution for 15 min, and then the NPC was placed at room temperature with 0.5% permeable Triton X-100 for 20 min. Wash with PBS and block with 5% goat serum. Collagen II (A24334, ABclonal, 1:400, Wuhan, China) and MMP-3 (A1202, ABclonal, 1:400) were incubated at 4°C overnight and washed with PBS for 3 times for 5 min each time. The cells were incubated with fluorescent secondary antibody at 37°C for 1 h, stained with DAPI for 3 min, and the images were analyzed under fluorescence microscope (Axio Vert A1, Carl Zeiss, Germany).

### 2.11. Apoptosis Detected by Flow Cytometry

P3-generation NPC was inoculated with 6 × 10^5^/well and cultured in 6-well plates until the cell fusion reached 80%. The cells were collected after different interventions according to the group. The Annexin V-FITC kit (C1062M, Beyotime, Shanghai, China) was used for flow cytometric detection of apoptotic cells. After digestion and centrifugation, the cells were rehung in 500 μL PBS, fully blown, added with 5 μL Annexin V-FITC solution, incubated for 15 min in dark room temperature, then added with 5 μL PI staining solution, reacted for 5 min in dark room temperature, and then tested by machine.

### 2.12. Animals and Experimental Design

A total of 24 male SD rats purchased from SPF (Beijing) Biotechnology Co. Ltd were randomly divided into normal control group (NC group), degeneration control group (DC group), and PEG-CeO_2_NPs treatment group. No special treatment was performed in the NC group. In the remaining group, the 8th and 9th coccygeal vertebrae were marked, and 2% pentobarbital (40 mg/kg) was injected intraperitoneally, the skin at the puncture site was disinfected, and 21G needles were used to penetrate the vertebral space vertically. Rotate the needle 360° and hold for 30 s. Immediately after the establishment of the model, the PEG-CeO_2_NPs treatment group was injected with 3 μg (0.01 mg/kg) of PEG-CeO_2_NPs, and the same amount of 3 μL PBS was injected locally into the same segment of the DC group. After IVDD puncture modeling, we observed the behavior of the animals to assess the degree of pain, kept the rats in a single cage to protect them from attack, and used soft bedding, high-calorie feed, and free drinking water to promote postoperative recovery of the animals. Over the next 3 days, all animals were injected subcutaneously with 0.03 mg/kg buprenorphine for postoperative analgesia. Four weeks after surgery, all rats were examined by X-ray and magnetic resonance imaging (MRI) before death, and then Co8-9 intervertebral disc specimens were taken for tissue sections. All animal operations in this study were carried out in accordance with the Guidelines for the Care and Use of Laboratory Animals of the National Institutes of Health of China and the Working Procedures of the Working Group Committee for the Ethical Review of Laboratory Animal Welfare of Tongji University. This study was approved by the Ethics Committees of Shanghai East Hospital, Tongji University School of Medicine (Approval No. EC. D (BG).016.02.1).

### 2.13. X-Ray Film and MRI Analyses

Radiographs (uDR 588i, United Imaging, Shanghai, PR) were used in each group to evaluate the vertebral space height, and the disc height loss was evaluated using the disc height index (DHI). DHI% = (*D* + *E* + *F*) × 100%/(*A* + *B* + *C* + *D* + *E* + *F*) [14]. In this calculation formula, *A* is the height of the anterior margin of the upper vertebral body, *B* is the height of the midpoint of the upper vertebral body, and *C* is the height of the posterior margin of the upper vertebral body. *D* is the height of the anterior margin of the intervertebral space, *E* is the height of the midpoint of the intervertebral space, and *F* is the height of the posterior margin of the intervertebral space.

3.0T MRI (Philipps-Achieva 3.0T, The Netherlands) was used to evaluate the positive effect of PEG-CeO_2_NPs on intervertebral discs in vivo. The signal strength of the disc was analyzed using axial T2-weighted MRI, and the degree of IVDD was assessed in a double-blind manner by Pfirrmann grading. The Pfirrmann grading is divided into five levels, from Grade I to Grade V [15].

### 2.14. Histological Analysis

4 weeks after surgery, the rats were injected intraperitoneally with excess pentobarbital and Co8-9 fragments were removed. The disc specimens were fixed in 4% Paraformaldehyde Fix Solution (G1101-500ML, Servicebio, Wuhan, China), decalcified with EDTA Decalcification Solution (G1105-500ML, Servicebio, Wuhan, China), dehydrated with alcohol, and embedded in paraffin wax. Finally, the sample was cut into a thickness of 5 μm and fixed to the slide with a neutral resin. H&E staining and Safranin O-Fast Green staining were used to observe the morphology of AF, NP boundary, and NPC. Images were collected using a digital sliding scanner (C1323901, Hamamatsu, Japan). Referring to the scoring criteria in previous studies [16], histological scores (0–12) were assigned to evaluate the degree of IVDD, as shown in Table 1.

### 2.15. Biosafety Assessment In Vivo

After the experimental rats were euthanized, the heart, liver, spleen, lung, and kidney tissue samples of NC group, DC group, and PEG-CeO2NPs treatment group were fixed in 4% Paraformaldehyde Fix Solution, embedded in paraffin, cut into 5 μm thick slices, and then H&E staining was performed to evaluate the effects of PEG-CeO2NPs on the major organs of the body.

### 2.16. Statistical Analysis

SPSS 25.0 statistical software was used to analyze the data, and GraphPad Prism 9 was used to make statistical charts. When the data of the two groups appeared, the results were significant (*p* < 0.05). The Tukey multiple comparison test was then used to further assess the differences between the groups.

## 3. Results

### 3.1. Characterization and Activity of PEG-CeO_2_NPs

**Figure 1:**
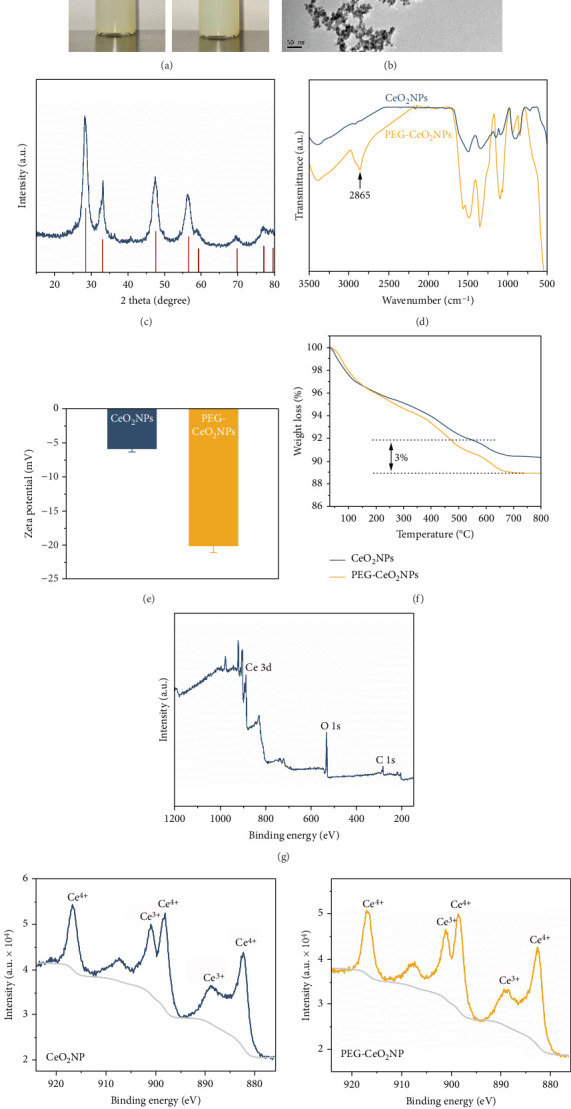
Morphology and composition of PEG-CeO_2_NPs. (a) Optical images of PEG-CeO_2_NPs. (b) Microscopic image of PEG-CeO_2_NPs observed by TEM. (c) XRD pattern of PEG-CeO_2_NPs. FTIR spectra (d), Zeta potentials, (e) and TGA curves (f) of CeO_2_NPs and PEG-CeO_2_NPs. (g) XPS spectrum of PEG-CeO_2_NPs. (h) High-resolution XPS spectra of Ce 3d for CeO_2_NPs and PEG-CeO_2_NPs.

The as prepared PEG-CeO_2_NPs were dispersed and stored in deionized water. After 14 days of static settling, the macroscopic dispersion state of the NPs is shown in Figure 1(a). The NP solution still maintained a uniform state without significant stratification, indicating an excellent dispersion stability in the aqueous solution. A deeper understanding of the formation process of PEG-CeO_2_NPs would allow us to interpret the effect of PEG modification. In aqueous solution, cerium ion will hydrolyze and form complex precursors as intermediates, which can turn into CeO_2_NPs in alkaline environment. In PEG solution, the ether and hydroxyl groups in PEG molecular chains will coordinate with cerium ion in the complexation reaction, facilitating the nucleation and growth of nanocrystals [17]. Meanwhile, after the initial formation, CeO_2_ nanocrystals are quickly covered by PEG, limiting their further growth and aggregation. In addition, the spatial repulsive forces between the PEG layers can reduce the van der Waals attraction between the NPs to some extent, thus enhancing the dispersion stability of the NPs.

The micromorphology of PEG-CeO_2_NPs was observed by TEM (Figure 1(b)), showing a well-dispersed state with an average particle size of about 5–10 nm.  Figure 1(c) presents the XRD pattern of PEG-CeO_2_NPs. The strong characteristic peaks at 2*θ* = 28.55, 33.08, 47.48, and 56.34 correspond to the (111), (200), (220), and (311) crystal faces of CeO_2_ (PDF#43-1002). PEG modification was characterized by FTIR analysis (Figure 1(d)). The new characteristic absorption peak at 2865 cm^−1^ attributed to the stretching vibration of the -CH_2_ groups in the PEG molecular chain [18].  Figure 1(e) demonstrates the surface electronegativity of CeO_2_NPs and PEG-CeO_2_NPs. Due to the large number of hydroxyl groups in the molecular chain, the charge of PEG is negative in aqueous solution. The significant decrease in Zeta potentials of the NPs from −5.84 ± 0.50 mV to −20.15 ± 1.01 mV suggested the successful modification of PEG. On this basis, thermal decomposition behavior of CeO_2_NPs and PEG-CeO_2_NPs was investigated by TGA at 30°C–800°C (Figure 1(f)). The curve showed that the mass loss of the NPs mainly occurred in two temperature regions. The first region between 100°C and 300°C indicated an initial weight loss, possibly related to the adsorbed water on the surface [19]. Between 400°C and 700°C, CeO_2_NPs showed no significant weight loss, while PEG-CeO_2_NPs exhibited a secondary mass loss plateau, which could be attributed to the decomposition of the surface PEG [20]. The content of surface PEG was approximately 3%. XPS was utilized to analyze the elemental composition and chemical states of the NPs (Figures 1(g) and 1(h)). The XPS survey spectra indicated that PEG-CeO_2_NPs were composed of Ce, O, and a simple organic modification layer. High-resolution XPS spectra of Ce 3d showed a mixture of Ce^3+^ and Ce^4+^ valence states in both CeO_2_NPs and PEG-CeO_2_NPs, providing a chemical basis for their catalytic activities.

**Figure 2:**
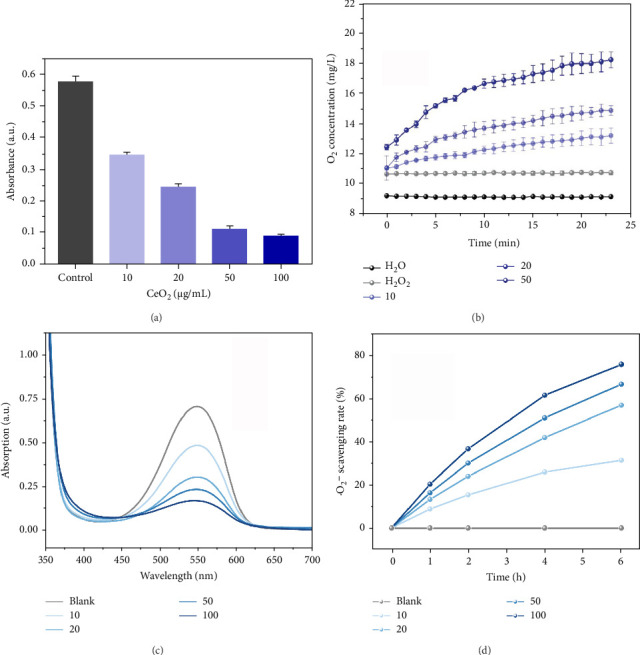
The ROS scavenging ability of PEG-CeO_2_NPs. The remaining content of H_2_O_2_ (a), the O_2_ generation rate in H_2_O_2_ (b), the remaining content of •O_2_^−^ (c), and •O_2_^−^ scavenging rate under different reaction duration (d) with different PEG-CeO_2_NPs concentration treatment.

The ability of PEG-CeO_2_NPs to scavenge two common ROS, H_2_O_2_ and superoxide anion (•O_2_^−^), was evaluated. The H_2_O_2_ scavenging capacity of PEG-CeO_2_NPs at different concentration gradients was investigated.  Figure 2(a) shows the absorbance of the samples at the wavelength of 200 nm which corresponded to the major adsorption peak of H_2_O_2_. It was found that the absorbance of the remaining H_2_O_2_ at 200 nm gradually decreased with the increasing concentration of PEG-CeO_2_NPs, indicating a proportional relationship between the H_2_O_2_ scavenging activity and concentration. Due to the generation of oxygen during the catalytic decomposition of H_2_O_2_, the concentration of dissolved oxygen was monitored (Figure 2(b)). The oxygen in deionized water remained stable at a low level, while in the H_2_O_2_ control group, the oxygen concentration slightly increased because of the natural self-decomposition of H_2_O_2_, with a relatively stable oxygen generation rate over time. When PEG-CeO_2_NPs were added into H_2_O_2_, the oxygen concentration in the solution continuously increased with prolonged reaction time. Furthermore, the oxygen generation rate enhanced with the increase of PEG-CeO_2_NPs concentration, consistent with the above conclusion. Figures 2(c) and 2(d) illustrate the •O_2_^−^ scavenging capacity of PEG-CeO_2_NPs. Similarly, the solution with a higher concentration of PEG-CeO_2_NPs exhibited a weaker absorbance near 550 nm, indicating the good •O_2_^−^ scavenging activity of PEG-CeO_2_NPs in a dose-dependent manner. In addition, the •O_2_^−^ scavenging rate increased with the extension of reaction time. After 6 h, the amount of scavenging·•O_2_^−^ reached significantly 75.67% with the PEG-CeO_2_NPs concentration of 100 μg/mL. These results confirmed that PEG-CeO_2_NPs exhibited both excellent H_2_O_2_ and •O_2_^−^ scavenging activity, holding the potential to eliminate excess ROS under oxidative stress and delay the degeneration of intervertebral disc.

### 3.2. PEG-CeO_2_NPs Decreased ROS Levels and ROS-Induced Apoptosis in NPCs

**Figure 3:**
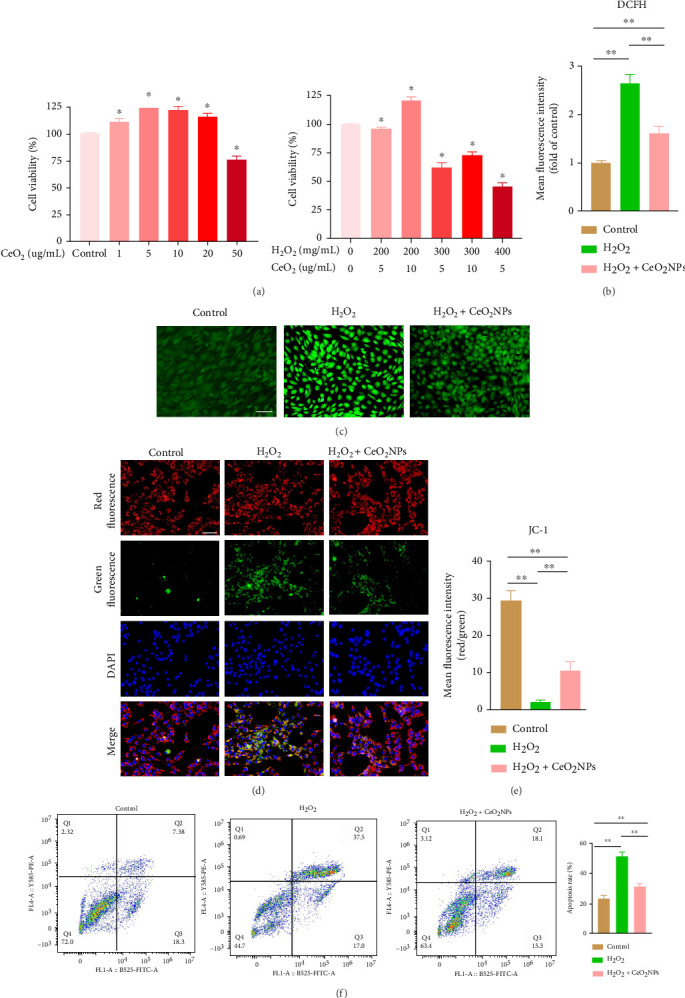
PEG-CeO_2_NPs decreased ROS levels and ROS-induced apoptosis in NPC. (a) Cell viability of different concentrations of PEG-CeO_2_NPs and different concentrations of H_2_O_2_ + PEG-CeO_2_NPs incubated with NPC for 24 h. (b, c) Representative fluorescence imaging and mean fluorescence intensity of intracellular ROS assessed by DCFH probe (*n* = 3). Scale bar = 50 μm. (d, e) Representative fluorescence images of mitochondrial potentials measured by JC-1 staining and mean fluorescence intensity (*n* = 3). Red: JC-1 aggregates; they reflect a higher mitochondrial membrane potential. Green: JC-1 monomers; they reflect a lower mitochondrial membrane potential, and JC-1 cannot accumulate in the matrix of the mitochondria. Scale bar = 50 μm. (f) Annexin V/PI staining and flow cytometric indication analysis to assess the percentage of apoptotic cells (*n* = 3). One-way ANOVA and Tukey's multiple comparisons test were used for statistical analysis. ^∗^Indicates that the data of each group were statistically different from that of the control group, and *p* < 0.05; ^∗∗^indicates that there are statistical differences among the groups, and *p* < 0.01.

In order to determine the effect of PEG-CeO_2_NPs on the viability of NPCs, we conducted a CCK8 experiment (Figure 3(a)), which showed that PEG-CeO_2_NPs had no inhibitory effect on the viability of NPC when the concentration was lower than 20 μg/mL. H_2_O_2_ was used to simulate oxidative stress in NPC [21]. When the concentration of H_2_O_2_ was 200 mg/mL, PEG-CeO_2_NPs of 10 μg/mL could still maintain a high vitality of cells. Therefore, PEG-CeO_2_NPs with a concentration of 10 μg/mL were selected for the subsequent experiment. DCFH-DA is a cell permeability probe used to detect the concentration of H_2_O_2_ or superoxide in cells [22]. DCFH probe imaging results showed that PEG-CeO_2_NPs reduced intracellular ROS concentration after H_2_O_2_ treatment (Figures 3(b) and 3(c)). When ROS levels are too high, abnormal mitochondrial membrane potential will result in an increase in intrinsic apoptosis [23, 24]. Therefore, we studied whether PEG-CeO_2_NPs can regulate mitochondria to return to normal potential and reduce cell apoptosis by clearing ROS. Mitochondrial membrane potential was measured (Figures 3(d) and 3(e)), and the results showed that the red fluorescence decreased in the H_2_O_2_ treatment group (200 mg/mL), while the green fluorescence increased, indicating that the mitochondria changed from a polarized state to a depolarized state under H_2_O_2_, and the mitochondria became unhealthy. PEG-CeO_2_NPs partially reverse this effect. We subsequently verified by flow cytometry that PEG-CeO_2_NPs reduced the percentage of apoptotic NPC under oxidative stress (Figure 3(f)). These results suggest that PEG-CeO_2_NPs can reduce ROS levels and ROS-induced apoptosis in NPC cells.

### 3.3. PEG-CeO_2_NPs Can Resist Cell Senescence and Cell Proliferation Damage Caused by ROS

**Figure 4:**
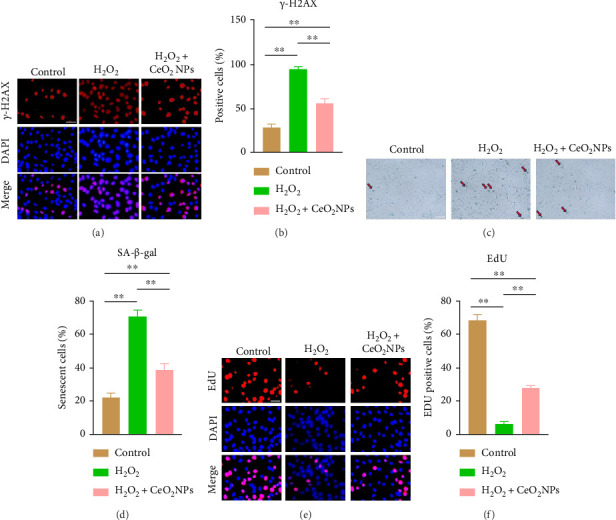
PEG-CeO_2_NPs can resist cell senescence and cell proliferation damage caused by ROS. (a, b) Representative immunofluorescence image of γ-H2AX in NPC. Scale bar = 50 μm. Semi-quantitative analysis of fluorescence intensity (*n* = 3/group). (c) NPC SA-β-gal staining representative images. Arrows indicate typical senescent nucleus pulposus cells. Scale bar = 100 μm. (d) Use ImageJ software to calculate the percentage of senescent cells (*n* = 3/group). (e) The proliferation rate of NPC was measured by EdU method. Scale bar = 20 μm. (f) Use ImageJ software to calculate the percentage of EdU positive cells (*n* = 3/group). One-way ANOVA and Tukey's multiple comparisons test were used for statistical analysis. ^∗∗^Indicates that there are statistical differences among the groups, and *p* < 0.01.

Cell senescence is a response to the accumulation of unrepaired damage in cells and is a key factor in the occurrence of degenerative diseases [2, 25, 26]. Excessive ROS levels will accelerate the accumulation of mitochondrial oxidative byproducts, resulting in decreased mitochondrial activity, multiple organelle dysfunction, and cell senescence [27, 28]. We have previously demonstrated that mitochondrial dysfunction occurs under H_2_O_2_ treatment, and PEG-CeO_2_NPs can partially reverse this effect (Figures 3(d) and 3(e)). H2AX (H2A histone family member X) is a variant of the histone protein H2A. When the cell DNA double strand breaks, Serine 139 on H2AX is phosphorylated to produce γ-H2AX [29]. H2AX staining has been widely used in the study of DNA damage and apoptosis, becoming an important marker of DNA damage. Through immunofluorescence staining, we found that NPC showed obvious DNA damage induced by H_2_O_2_, while PEG-CeO_2_NPs could resist oxidative stress damage induced by H_2_O_2_ on cells (Figure 4(a)). SA-β-gal is a biomarker of senescent cells [30]. We found that the level of β-galactosidase in the PEG-CeO_2_NPs + H_2_O_2_ group was significantly reduced compared with that in the H_2_O_2_ group (Figure 4(b)). EdU staining is a staining method used to measure cell proliferation activity [31]. We found that compared with H_2_O_2_ group, the proportion of cells with proliferation activity in PEG-CeO_2_NPs + H_2_O_2_ group increased, indicating that PEG-CeO_2_NPs could partially reverse the inhibitory effect of ROS on cell proliferation activity. These results suggest that PEG-CeO_2_NPs can resist cell senescence and cell proliferation damage caused by ROS.

### 3.4. PEG-CeO_2_NPs Improved the Anabolic and Catabolic Balance of NPCs

**Figure 5:**
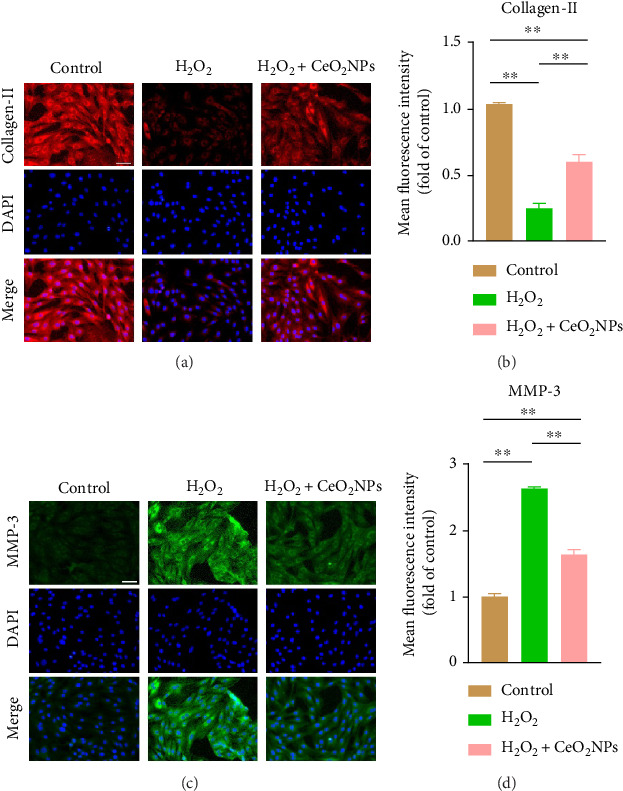
PEG-CeO_2_NPs improved the anabolic and catabolic balance of NPC. (a–d) Representative immunofluorescence images of collagen II and MMP-3 in NPC and semi-quantitative analysis of fluorescence intensity (*n* = 3/group). Scale bar = 50 μm. One-way ANOVA and Tukey's multiple comparisons test were used for statistical analysis. ^∗∗^Indicates that there are statistical differences among the groups, and *p* < 0.01.

Under normal physiological conditions, NPC can produce ECM components and maintain the dynamic balance of anabolism and catabolism. When the balance is broken, tissue decomposition increases, accelerating the occurrence of IVDD. At this time, the protein content of type II collagen synthesized by ECM decreased, and the expression of MMPs increased [32]. H_2_O_2_-induced oxidative stress can stimulate NPC to increase the expression of catabolic protein and reduce the level of anabolic metabolism, which can be manifested as a decrease in Col-II expression and an increase in MMP-3 expression [16]. We found that a large number of well-shaped spindle cells with Col-II staining positive were observed in the control group (Figures 5(a), 5(b), 5(c), and 5(d)), while MMP-3 staining was almost negative. In the H_2_O_2_ treatment group, the fluorescence intensity of Col-II staining decreased significantly, while that of MMP-3 positive staining increased significantly. However, in the H_2_O_2_+PEG-CeO_2_NPs treatment group, the fluorescence intensity of Col-II was restored significantly, while the green fluorescence of MMP-3 was decreased. These results indicate that PEG-CeO_2_NPs promote the restoration of the anabolic and catabolic balance of NPC.

### 3.5. The Level of IVDD Was Reduced in Rats Injected With PEG-CeO_2_NPs

**Figure 6:**
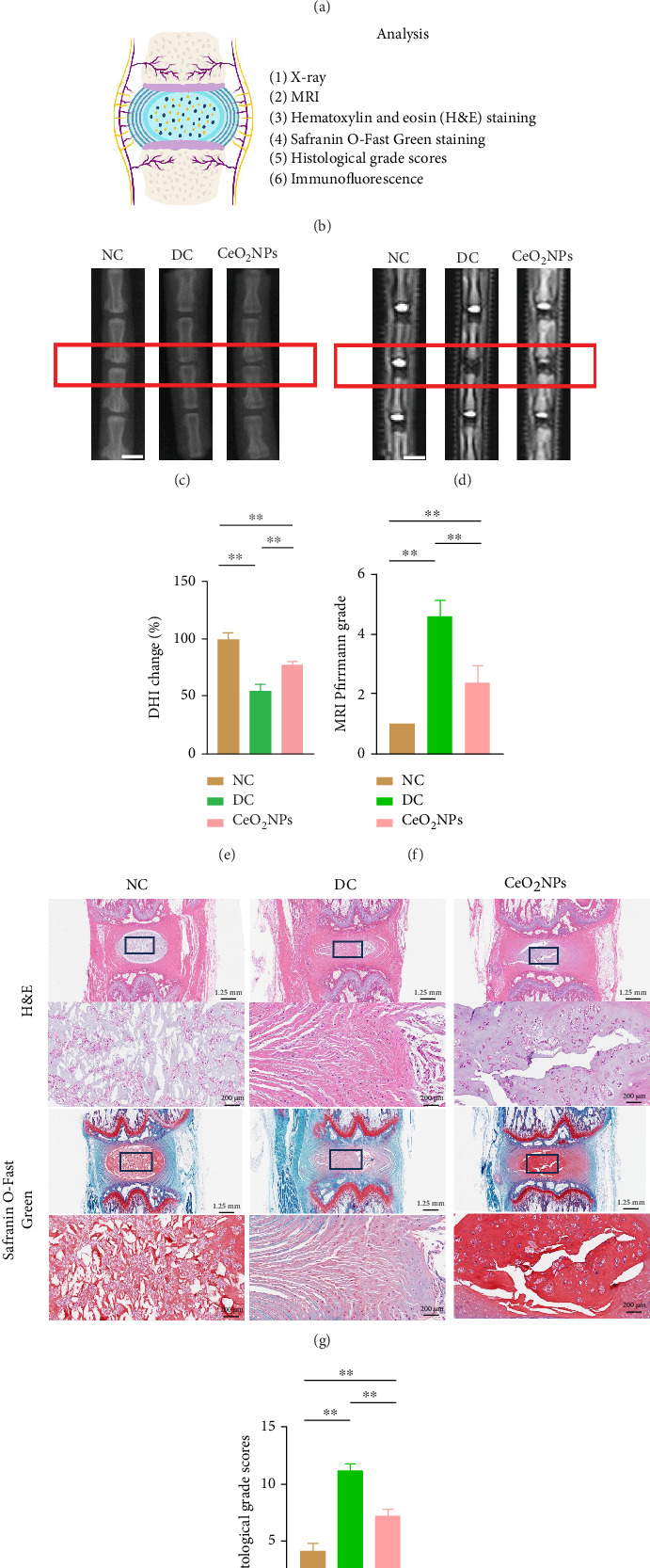
The level of IVDD was reduced in rats injected with PEG-CeO_2_NPs. (a) Schematic diagram of rat IVDD model construction. (b) Experimental design to evaluate IVDD degree after sampling. (c) X-ray showed the vertebral space height in the NC group, DC group, and CeO_2_NPs treatment group. Scale bar = 200 mm. (d) MRI showed the degree of disc degeneration in each treatment group. Scale bar = 200 mm. (e) Quantitative analysis of DHI% of each treatment group by ImageJ (*n* = 8/group). (f) Quantitative analysis of MRI Pfirrmann grading in each treatment group (*n* = 8/group). (g) Hematoxylin and eosin staining and Safranin O-Fast Green staining of each treatment group and local amplification of key parts; the larger area has a scale of 1.25 mm and the smaller area has a scale of 200 μm. (h) Quantitative analysis of histological grade scores in each treatment group (*n* = 8/group). One-way ANOVA and Tukey's multiple comparisons test were used for statistical analysis. ^∗∗^Indicates that there are statistical differences among the groups, and *p* < 0.01. NC: normal control group; DC: degeneration control group; CeO_2_NPs: PEG-CeO_2_NPs treatment group.

In order to further understand the effects of PEG-CeO_2_NPs on IVDD in vivo, we constructed a rat IVDD model induced by acupuncture (Figure 6(a)). The detailed construction process is described in Materials and Methods. Imaging and histological evaluation are reliable indicators of disc degeneration (Figure 6(b)) [33–35]. As can be seen on X-ray examination, the discs in the NC group showed a higher vertebral space. In the DC group, the vertebral space height decreased at the corresponding site, while the PEG-CeO_2_NPs treatment group recovered the vertebral space height and significantly increased DHI% compared to the DC group (Figures 6(c) and 6(e)). MRI can show the water content of the intervertebral disc, and the water content of NP is indicated by the strength of the T2-weighted signal. The NC group showed bright MRI images of the NP, while the T2-weighted signal at the corresponding part of NP in the DC group was significantly reduced, consistent with the X-ray results, and the T2-weighted signal at NP in the PEG-CeO_2_NPs treatment group was restored significantly (Figure 6(d)). MRI Pfirrmann grading was much lower in the PEG-CeO_2_NPs group than in the DC group, indicating that the degree of IVDD was reduced in the PEG-CeO2NPs group compared with the DC group (Figure 6(f)). From the imaging perspective, PEG-CeO_2_NPs did reduce needle-induced IVDD in rats.

**Figure 7:**
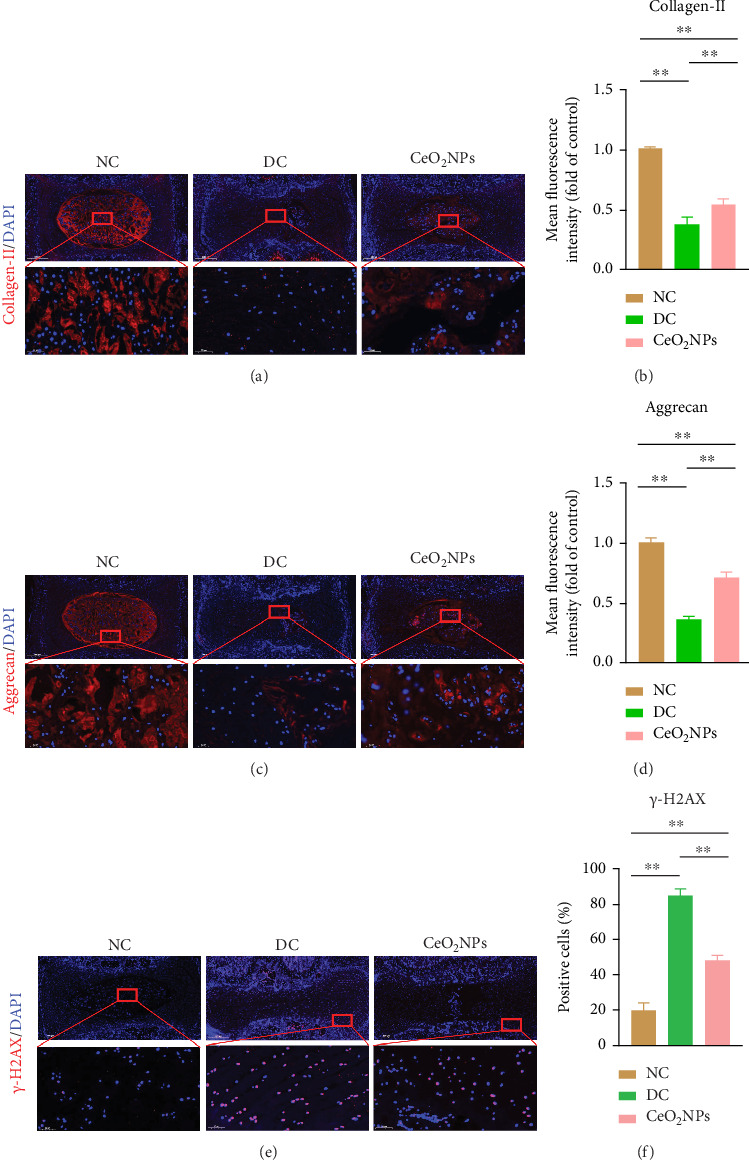
PEG-CeO_2_NPs correct synthesis and catabolic disorders of ECM in rats. (a, b) Representative immunofluorescence images of collagen II and semi-quantitative analysis of fluorescence intensity of animal intervertebral disc slices of each treatment group (*n* = 8/group). (c, d) Representative immunofluorescence images of MMP-3 and semi-quantitative analysis of fluorescence intensity of animal intervertebral disc slices in each treatment group (*n* = 8/group). (e, f) H2AX representative immunofluorescence images and semi-quantitative analysis of fluorescence intensity of animal intervertebral disc samples in each treatment group (*n* = 8/group). The larger area has a scale of 500 μm and the smaller area has a scale of 50 μm. One-way ANOVA and Tukey's multiple comparisons test were used for statistical analysis. ^∗∗^Indicates that there are statistical differences among the groups, and *p* < 0.01. NC: normal control group; DC: degeneration control group; CeO_2_NPs: PEG-CeO_2_NPs treatment group.

Histological slides were obtained 4 weeks after acupuncture and injection of PEG-CeO_2_NPs. H&E staining showed that the disc structure in the NC group was normal, the disc in the DC group showed a degenerative phenotype, the disc height was lower than that in the NC group, and the boundary between the NP and the AF was unclear. The NP and AF in the PEG-CeO_2_NPs treatment group were almost in normal structure, significantly better than those in the DC group (Figure 6(g)). In addition, S-O staining results showed that the structure of NP and AF was disordered in the DC group. The disordered arrangement of AF led to unclear boundaries and accelerated ossification of cartilage. This resulted in disc collapse and fusion. The PEG-CeO_2_NPs treatment group significantly reversed the morphology of IVDD (Figure 6(g)). These results are consistent with the above H&E staining results. Histological scores showed that the score of PEG-CeO_2_NPs treatment group was significantly lower than that of DC group (Figure 6(h)). Immunofluorescence results showed that the fluorescence intensity of collagen II in the DC group decreased significantly compared with that in the NC group, which was consistent with our expected results, while the PEG-CeO_2_NPs treatment group could recover the decrease in collagen II content caused by IVDD (Figures 7(a) and 7(b)). A similar trend was shown in Aggrecan immunofluorescence images (Figures 7(c) and 7(d)), indicating that PEG-CeO_2_NPs can improve extracellular matrix synthesis and catabolic disorders caused by IVDD. As mentioned earlier, H2AX is a marker of DNA damage that reflects the aging state of cells. H2AX immunofluorescence staining showed that the proportion of senescent cells in the DC group was significantly higher than that in the NC group, while the PEG-CeO2NPs treatment group could reverse the senescent trend of the DC group and restore the cell state (Figures 7(e) and 7(f)). X-ray, MRI image measurement, H&E staining, S-O staining, and immunofluorescence showed that the degree of IVDD in PEG-CeO_2_NPs treatment group was significantly recovered compared with that in DC group. In summary, the in vivo study further demonstrated the feasibility of local injection of PEG-CeO_2_NPs to reverse the progression of IVDD.

### 3.6. Safety Assessment of PEG-CeO_2_NPs

**Figure 8:**
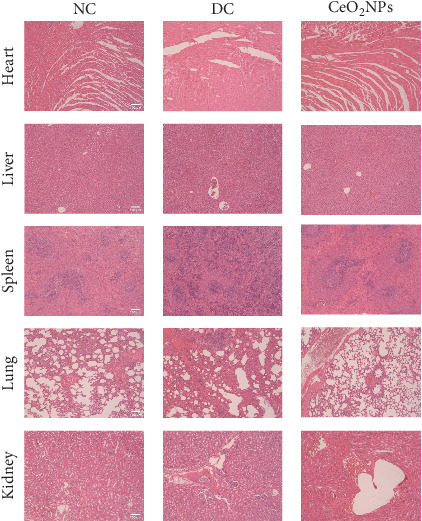
H&E-stained sections of major organs. NC: normal control group; DC: degeneration control group; CeO_2_NPs: PEG-CeO_2_NPs treatment group.

Although our experimental results show that PEG-CeO_2_NPs have good ROS clearance ability, their metabolic pattern and their effects on the organism will require longer studies to prove. Currently, studies have shown that oral cerium oxide has no effect on human health under conditions based on mouse models [36]. When administered intravenously, more than 90% of PEG-CeO_2_NPs accumulate in the liver and spleen, with small amounts in the lungs and kidneys [12]. In this study, although not administered intravenously, we wanted to observe the safety of direct injection of PEG-CeO_2_NPs into the intervertebral disc. The safety assessment of PEG-CeO_2_NPs was evaluated with a dose of 0.01 mg/kg. H&E staining was performed on major organs of rats at 8 weeks, and it was found that after intervertebral disc injection of PEG-CeO_2_NPs, no lesions were found in major organs of rats in the acupuncture group and the NC group (Figure 8). It was demonstrated that PEG-CeO_2_NPs had no obvious side effects on organisms when injected directly into intervertebral disc.

## 4. Discussion

Under normal conditions, the total amount of intracellular ROS remains dynamically stable [37]. However, when senescence occurs, the scavenging ability of SOD and CAT in cells decreases, resulting in the accumulation of ROS, which leads to a series of adverse events. Cell senescence is the accumulation of cell damage to a certain extent and irreversible cell cycle stagnation [26], which forms a vicious cycle. ROS is therefore a critical therapeutic target.

Intervertebral disc is the largest avascular structure in the human body [38], and NPC mainly obtains energy by anaerobic glycolysis. Compared with aerobic respiration, the ROS produced by electron transport chain and membrane peroxidation of mitochondria in this process may be relatively less [39]. However, IVDD is mostly an age-related degenerative disease. When ROS accumulates to a certain level within the cell, the homeostasis within the NPC is disrupted, and the excessive ROS causes irreversible damage to the NPC. A relatively severe DNA damage reaction will occur [40]. At this time, cell proliferation will stop, senescence-associated secretory phenotype (SASP) will be produced [41], and cells will produce excessive inflammation and catabolic factors. The anabolic and catabolic homeostasis of ECM are disrupted [42], resulting in a decrease in Col-II synthesis and an increase in MMP synthesis, which promotes the decomposition of ECM and further decreases the water retention capacity of ECM, thus showing a grain-black plane in MRI T2-weighted plane and decreasing the ability of intervertebral disc to buffer gravity and stress load. It causes lumbar disc herniation (LDH) and LBP.

Cerium oxide (CeO_2_) is a lanthanide element, and strong Ce3+ to Ce4+ REDOX couples help achieve considerable reducibility [43]. At the same time, CeO_2_ has many in-depth studies in liver, lung, eye, brain, and other organs due to its advantages of low cost, high efficiency, high stability, large production, and easy processing [12, 44–46]. Its broad-spectrum antioxidant ability can also be applied to dressing for trauma [11], and it is a nano-enzyme with good prospects for future transformation. In recent years, some studies are not limited to the direct injection of CeO_2_ or the use of CeO_2_ as a dressing, but the joint assembly of cerium oxide nano-drugs with other carriers, so as to achieve a more efficient effect, such as the joint assembly of cerium oxide NPs with human umbilical cord blood mesenchymal stem cells, so as to effectively resist oxidative stress. It can also use the dry nature of stem cells to promote tissue regeneration [43]. In addition, there have been studies on the assembly of cerium oxide NPs and siRNA into ultrasound-sensitive nano-bubbles, which can be controlled by ultrasound as a controllable platform and thus play a role in bone cells [47]. This gives us a lot of inspiration and helps to achieve better treatment of disc degeneration.

## 5. Conclusion

In summary, we prepared a novel cerium oxide nano-enzyme PEG-CeO_2_NPs, whose characterization and properties are superior to those of traditional antioxidants. At the same time, it has the characteristics of safety, reliability, easy preparation, and high efficiency. We verified that PEG-CeO_2_NPs can effectively clear excess ROS and correct NPC metabolic disorders in vitro and further verified its reversal of disc degeneration in rat disc acupuncture degeneration model, with significant effects, which may provide a new therapeutic idea for patients with cervical and lumbar degenerative diseases clinically.

## Figures and Tables

**Table 1:** Definition of histological grade. Reproduced from Dai et al., Mater Today Bio. 2022 (https://pmc.ncbi.nlm.nih.gov/articles/PMC9758573).

Category	Grade
I. Matrix of the nucleus pulposus	1. Normal gelatinous appearance
	2. Slight condensation of the extracellular matrix
	3. Moderate/severe condensation of the extracellular matrix

II. Cellularity of the nucleus pulposus	1. Normal cellularity with large vacuoles in the gelatinous structure of the matrix
	2. Slight decrease in the number of cells and fewer vacuoles
	3. Moderate/severe decrease (> 50%) in the number of cells and no vacuoles

III. Structure of annulus fibrosus	1. Normal, pattern of fibrocartilage lamellae without ruptured fibers and without a serpentine appearance anywhere within the annulus
	2. Slight ruptured or serpentined patterned fibers (< 30%)
	3. Moderate/severe ruptured or serpentined patterned fibers (> 30%)

IV. Border between the nucleus pulposus and annulus fibrosus	1. Normal
	2. Minimally interrupted
	3. Moderate/severe interruption

## Data Availability

The data that support the findings of this study are available from the corresponding authors upon reasonable request.
